# Effects of the Laser Micromelting Process Parameters on the Preparation of Micron-Sized FeCrAl Coatings on Zr Alloy Surfaces

**DOI:** 10.3390/ma16237421

**Published:** 2023-11-29

**Authors:** Guoqing Song, Wentian Wei, Botao Liu, Bincai Shuai, Gengming Liu, Kanghui Xue, Yong Chen

**Affiliations:** College of Mechanical Engineering, University of South China, Hengyang 421101, China

**Keywords:** FeCrAl coating, numerical simulation, temperature field analysis, laser micromelting, laser process parameters, surface morphology

## Abstract

Laser micromelting (LMM) technology allows for the remelting of pre-positioned coatings on the surface of a specimen to form a metallurgical bond with the substrate material, significantly improving the coating’s film–base bond. However, the high energy input from the laser modification process can cause severe element diffusion, rendering the coating susceptible to deformation and cracking. This can be mitigated by controlling the laser power, scanning speed, and offset of the LMM process. The temperature and stress fields of the samples in the LMM process were analyzed via finite element simulation. The effects of the LMM process parameters on the coating morphology were analyzed in conjunction with experiments. The results indicated that the laser power significantly affected the morphology of the coating after remelting, and a higher scanning speed was more likely to cause the coating to accumulate stress. Additionally, a smaller offset inhibited crack generation. At a laser power of 30 W, a scanning speed of 1200 mm/min, and a scanning spacing of 0.035 mm, the surface of the coating had no obvious defects and was relatively flat, and the adhesion and corrosion resistance were significantly improved. This study provides valuable guidance for improving the preparation of micron-sized protective coatings on Zr alloy surfaces.

## 1. Introduction

FeCrAl coatings have excellent resistance to high-temperature oxidation; they can produce dense Cr_2_O_3_, Al_2_O_3,_ and Fe_2_O_3_ oxidized films under high-temperature environments, blocking the oxygen entry channel, and are considered to be among the best choices for nuclear Zr alloy surface-protection coatings [1,2,3,4,5,6,7,8]. The protective coating on the surface of Zr cladding tubes is usually around 20 μm due to the limitations of the neutron absorption cross-section of the material and the assembly accuracy requirements [9,10,11]. Due to the thinness of the coatings prepared on the surfaces of Zr alloys, physical vapor deposition (PVD) technology is commonly utilized. PVD technology allows for the deposition of a wider range of materials including metals, alloys, and ceramics, and, more importantly, allows for precise control of the thickness of the coating deposited. However, because of the characteristics of PVD equipment and technology, when ferromagnetic materials are deposited, the metal droplets evaporated by the arc are reabsorbed into the target and the surface of the protective tiles, resulting in the short-circuiting of the equipment circuits or even their overloading and burnout. Electroplating technology can also be used to prepare micron-sized coatings; however, the coating preparation process is subject to external interference [12,13]. Coatings prepared using either method have the disadvantages of low binding strengths and high porosity, and they tend to fall off when exposed to shock loads [14,15,16,17]. Laser surface modification technology is prone to causing deformation of the workpiece and has a low assembly precision of the processed parts due to its high heat input. LMM technology can effectively reduce and control the laser energy deposition density to realize the modification of ultra-thin coatings. Therefore, prepared coatings are modified via LMM technology to improve their chemical and mechanical properties. The LMM treatment allows the pre-positioned coating to remelt and bond with the substrate, significantly improving its film–base bonding and densification [18,19,20,21]. Numerous studies have indicated that laser power, scanning speed, and offset are the main process parameters affecting the quality of cladding coatings [22,23,24]. A single-variable control method was applied to investigate the effect of each of the three process parameters on the coating quality. Nevertheless, the temperature still varies significantly during laser processing. The thermophysical properties of the material exhibit nonlinear changes. It is difficult to accurately measure the temperature and stress distribution patterns during the laser scanning process. With the continuous development of computer technology, the solving efficiency of numerical simulations have increased, and accurate mathematical models in complex simulation environments have been widely used to study the temperature and stress involved in laser processing [25,26,27,28,29,30]. Therefore, the residual stresses in the coating after the LMM treatment were analyzed by combining them with a simulation.

The current protective coatings prepared on Zr alloy surfaces have the problem of low adhesion. Here, CrAl and Fe coatings pre-deposited on Zr alloys via PVD and electroplating were treated using LMM technology. The aim is to obtain a composite metal protective coating with high adhesion and precisely controllable thickness. Through this composite technology, the problems of the ferromagnetic material not being preparable via PVD technology and the non-uniformity of the coating prepared via electroplating technology are solved, and the adhesion force of the coating is greatly improved. The precise preparation of the composite metal coating with a controllable thickness and high adhesion at the micron level is realized. Ansys software (2020 R2) was used to simulate the temperature field and stress field in the LMM process, which solved the problem of undetectable changes in the amount of melting and stress between the coating and the substrate during laser processing. Combined with the experimental results, the effects of different process parameters on the coating quality were examined. A composite metal protective coating with high adhesion was successfully prepared by optimizing the laser processing process parameters. An improved strategy is provided for the preparation of composite metal protective coatings with high adhesion on Zr alloy surfaces.

## 2. Methodology

### 2.1. Materials and Methods

A Zr alloy sheet (Zr-1Nb) was used in the experiments, which was processed into a standard specimen with dimensions of 20 × 20 × 5 mm^3^ via wire-cut electrical discharge machining (WEDM). The purity of the CrAl alloy target (Beijing Hezong Technology Co., Ltd., Beijing, China) was 3N (Cr:Al = 73:27 at.%). The surface of the Zr alloy was sanded with 2000# sandpaper, ultrasonically cleaned with alcohol for 30 min, and rinsed with plasma-treated water. Finally, the sample was placed in a drying oven at 50 °C for 20 min to prepare the coating. A TSU-650 multifunctional coating machine (Beijing Technol Co., Ltd., Beijing, China) was used. Details regarding the deposition processes are presented in Table 1. Electroplating experiments were performed using a deep eutectic solvent consisting of choline chloride-ethylene glycol (ChCl-EG) and a main salt of ferrous chloride (FeCl_2_·4H_2_O), whose compositions are presented in Table 2. The specimens were connected to the cathode, and the anode was treated with an insoluble titanium electrode, which was reversed for electrolytic activation before plating. A DDK-I test power supply (Shaoxing Chengtian Electric) was used in the experiments, and the parameters of the plating process are presented in Table 3. An XL-F300 fiber laser (Guangzhou Xinglai Laser Technology Co., Guangzhou, China) was used in the experiments, and the samples were preheated at 300 °C before LMM treatment.

The samples were polished via WEDM. The morphology of the samples was analyzed using HitachiSU-8000 SEM (Hitachi, Japan), and the cross-sectional elemental distributions were analyzed using energy-dispersive X-ray spectroscopy (EDS). The physical phase of the coatings was analyzed using an X-ray diffractometer (XD-3) with a Cu anode target. The scanning speed was 4°/min, and the scanning range was 2θ. The coating bonding force was analyzed using a WS-2005 coating adhesion automatic scratch tester. The scratch length was 5 mm, the loading speed was 50 N/min, and the termination load was 50 N. A CS2350H electrochemical workstation was used to test the corrosion resistance of the coatings, and the experimental corrosion solution used was a 3.5 wt.% NaCl solution. The salt bridge was prepared using 30 wt.% KCl and 3 wt.% agar powder in a heated water bath. The auxiliary electrode was a Pt conductivity electrode, and a saturated calomel electrode was used as the reference electrode.

### 2.2. Simulation and Calculation

**Figure 1:**
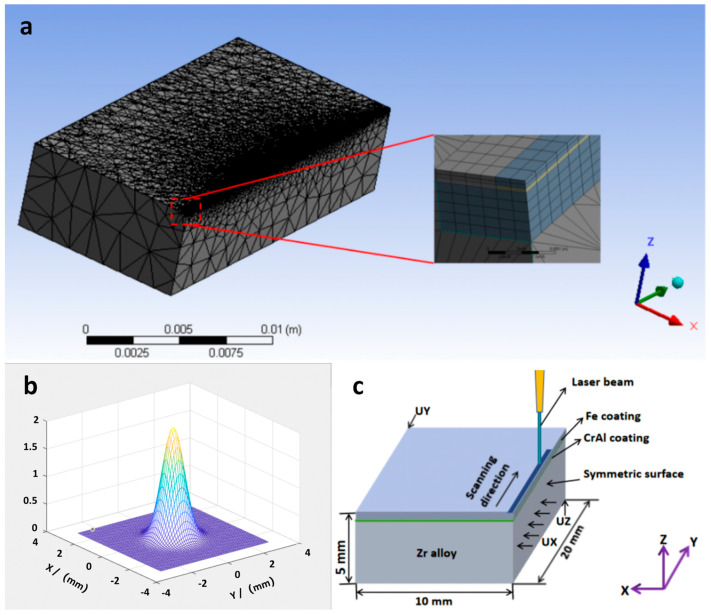
(**a**) Model meshing; (**b**) Gaussian heat source modeling diagram; (**c**) model boundary constraints diagram.

The variations in the temperature and stress fields during the LMM process were simulated using the Ansys software (2020 R2). Half of the samples were taken for modeling, where the thicknesses of the substrate and CrAl and Fe coatings were 5000, 7, and 13 μm, respectively. A symmetric model was created with a substrate size of 20 × 10 × 5 mm^3^ and a fused cladding layer size of 20 × 10 × 0.02 mm^3^. The mesh was refined for the heat-source scanning area and its vicinity, and a coarse mesh was used at a further distance from the heat source. The temperature-field fusion cladding layer used a refined hexahedral Solid90 elements, the substrate used tetrahedral Solid87 elements, and the surface effect element was Surf152. There were 93,089 elements and 247,807 nodes in total. The model meshing diagram is shown in Figure 1a.

A Gaussian surface heat source was used to simulate the LMM process, and the corresponding model is shown in Figure 1b. The expression is

(1) $$Q=\frac{2AP}{\pi R^{2}}\exp\{\frac{-2\cdot [{\left(x-x_{0}\right)}^{2}+{\left(y-vt\right)}^{2}]}{R^{2}}\}$$

where Q represents the heat flow density (W/m^2^) at the laser spot, *A* represents the absorption rate of the coating on the laser, *P* represents the output power of the laser (W), *R* represents the radius of the laser spot (m), *v* represents the scanning speed (m/s), *t* represents the scanning time (s), and (*x*_0_, *vt*) are the coordinates of the center of the spot at time *t*. 

The enthalpy method was used in the simulations for the latent heat of the phase change. The enthalpy H is expressed as

(2) H=∫ρc(T)dT

where *ρ* represents the material’s density (kg/m^3^), *c* represents the specific heat capacity of the material (J/Kg⋅°C), and *T* represents the temperature (°C).

According to the thermoelastic-plastic theoretical mechanical model, an indirect coupling method was used to analyze the stress field. Cladding was applied using refined hexahedral Solid186 units. The displacement degrees of freedom in the stress field were constrained using displacement, which restricts translational motion along the X-, Y-, and Z-axes, that is, UX, UY, and UZ. The constraint action areas in those three directions were selected as the peripheral and bottom surfaces of the geometric model, and the model boundary constraints are shown in Figure 1c. The thermophysical and mechanical property parameters of the material were simulated using JMatPro software (V 11.0), and the specific data are shown in Table 4 and Table 5.

## 3. Results and Discussion

**Figure 2:**
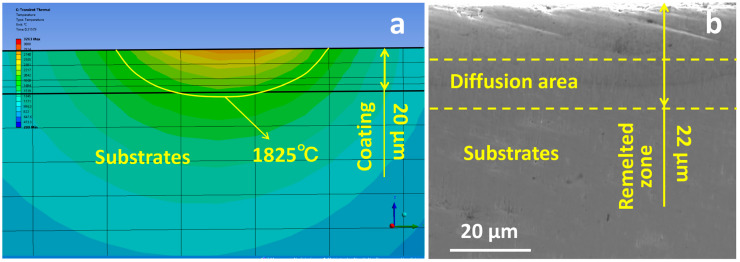
(**a**) Temperature field isotherm distribution of specimen cross-section; (**b**) cross-sectional morphology of specimen after LMM treatment.

The dilution rate of the substrate can intuitively respond to the performance of the molten layer; to illustrate the effect of different laser processing process parameters on the depth of the molten pool and metallurgical bonding, the expression of the substrate dilution rate is as follows:

(3) $$f=\frac{h}{h+H}$$

where *f* is the dilution rate, *h* is the depth of melting of the substrate (µm), and *H* is the melt pool depth (µm). The amount of substrate melting was judged based on the position of the depth of the substrate’s melting point isotherm (Figure 2a). After the coating was treated with LMM, the film–substrate demarcation line was not clear (Figure 2b). Under the same parameters (at a laser power of 30 W, a scanning speed of 1200 mm/min, and a scanning pitch of 0.035 mm), the actual melting depth of the specimen was about 22 µm, which was similar to the simulation results.

**Figure 3:**
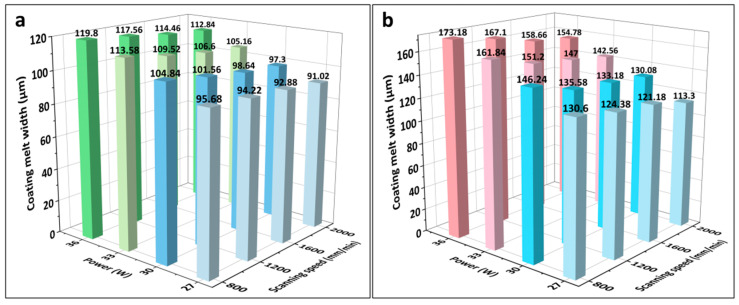

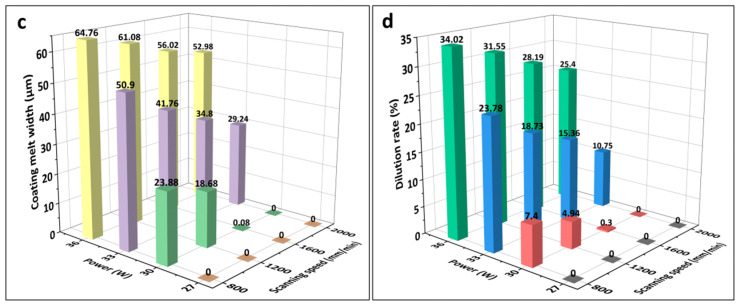
(**a**–**c**) Melting widths of the Fe and CrAl coatings and Zr substrate under different laser processes; (**d**) histograms of the dilution rate under different laser process parameters.

Figure 3 presents the melting widths of the specimens with different LMM process parameters. As shown, the melting widths of the coating and substrate increased with an increase in laser power and decreased with an increase in the scanning speed. As the laser power increased, the heat absorbed by the specimen increased and reached the melting point of the alloy, which started melting, whereas the laser power density abated when the scanning speed was too high. As shown in Figure 3c, at 27 W, the Zr substrate failed to melt, and the coating failed to form a metallurgical bond with the substrate. Although the Fe coating received laser irradiation directly and the CrAl coating obtained energy through heat conduction, the melting width of the CrAl coating exceeded that of the Fe coating in the same process. This was due to the good thermal conductivity of the material throughout the system and the lower melting point of CrAl compared with that of Fe. Due to the continuous dissipation of heat during the transfer process and the higher melting point of the Zr alloy compared to that of CrAl, the melting width of the Zr alloy matrix at the bottom of the system is far smaller than that of the CrAl coating. This allowed for the melting of wider Fe and CrAl coatings to be well-intercalated to form FeCrAl coatings. The narrower melting width of the Zr matrix prevented Zr from diffusing significantly into the coating because of excessive melting.

During laser processing, not only is a certain width of the melt pool required, but the depth of the melt pool must also exceed that of the coating to reach the Zr alloy substrate to form an effective metallurgical bond. However, it is necessary to prevent the Zr alloy substrate from melting excessively during the LMM process, which would lead to the diffusion of the Zr alloy into the coating surface and cause the coating to fail. As shown in Figure 3d, the laser power affected the substrate dilution rate and melt pool depth more significantly than the scanning speed. In addition, there was a significant difference in the substrate dilution rates for different processes with similar energy densities. For example, when the laser power is 27 W and the scanning speed is 1200 mm/min, and when the laser power is 36 W and the scanning speed is 1600 mm/min, the two energy densities are equal. However, in the former process, the substrate does not reach the melting point. While, in the latter process, the substrate melting depth is 7.85 μm. Higher power densities allow more heat to be absorbed at the location being lasered. Thereby, the temperature can be transmitted faster from the surface to the substrate. Therefore, the effect of the power density on the melting depth of the substrate is more significant than that of the energy density. Because of the microscale of the coating thickness, to prevent Zr diffusion to the surface the required substrate fusible depth is small, and the corresponding process parameter selection range is narrow. A laser energy density within the range of 11.25–22.5 J/mm^2^ gives a good dilution rate.

**Figure 4:**
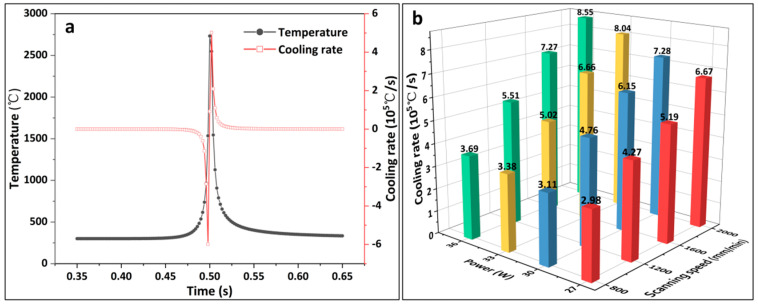
(**a**) Temperature and cooling rate of the midpoint of the scanning channel over time; (**b**) histogram of the maximum cooling rate of the midpoint of the scanning channel for different laser process parameters.

Figure 4a shows the variation curves of the temperature and cooling rate at the midpoint of the scanning channel for a laser power of 33 W and a scanning speed of 1200 mm/min. A negative cooling rate indicates a heating condition, whereas a positive cooling rate indicates a cooling condition. Analysis can be performed near the midpoint of the laser heat source; the warming and cooling rates gradually rise, and the temperature reaches a maximum value of 2732.4 °C when the laser scans to the midpoint position, while the temperature growth rate also reaches a maximum value. After the laser scanned past the midpoint, the temperature decreased and the positive cooling rate reached a maximum of 5.02 × 10^5^ °C/s. The laser then moved away from the midpoint; the temperature continued to decrease and the cooling rate decreased. It can be demonstrated that the LMM process exhibited the characteristics of rapid heating and cooling, and the non-equilibrium solidification characteristic unique to laser processing promotes the optimization of the microstructure. Simultaneously, the rapid heating and cooling increase the coating stress, such that the coating is prone to cracking. Figure 4b presents a histogram of the variation in the maximum cooling rate at the midpoint position for different process parameters. As shown, the cooling rate at the midpoint increased as the laser power and scanning speed increased. The scanning speed affected the cooling rate more significantly than the laser power. Laser processing occurs over a short period, and the cooling rate is too high to produce a high residual stress, resulting in cracks in the coating. To control the emergence of cracks in the coating, the laser power and scanning speed should be as low as possible while satisfying process requirements. However, an excessively low cooling rate is not conducive to tissue refinement and can degrade the overall mechanical properties of the coating [31].

**Figure 5:**
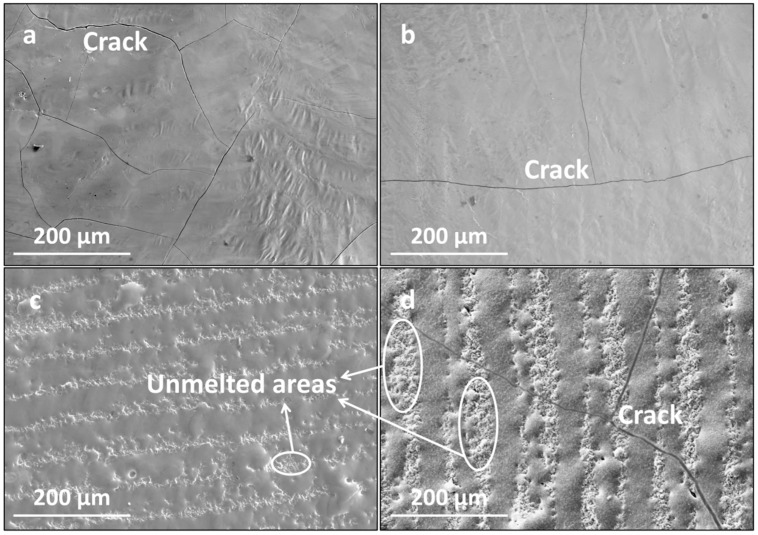
Surface morphologies of the coating at different scanning intervals: (**a**) 0.035 mm; (**b**) 0.045 mm; (**c**) 0.055 mm; and (**d**) 0.065 mm.

Figure 5 presents the surface morphologies of the LMM coatings at different scanning intervals. As shown, at smaller scanning intervals, the coating was flat after the LMM treatment, and the overlaps between the adjacent scanning lanes were good, but microscopic cracks were present. When the scanning spacing was increased, the overlap effect between neighboring scanning channels decreased, resulting in non-microfused areas between the scanning channels, which reduced the integrity and the densification of the coating. Meanwhile, the cracks widened. During the melting and solidification of the melt pool after the LMM treatment, a large amount of stress accumulated, and the adjacent scanning channel failed to form a heat-affected zone to release part of the stress. The mismatch of linear expansion coefficients between the different materials in the LMM process will cause deformation and stress concentration in the materials. The formula for the linear expansion coefficient *α* is as follows:

(4) $$\alpha =\frac{\Delta L}{L\times \Delta T}$$

where L is the expansion size (μm) and T is the temperature (°C). The strain transfer caused by the expansion and contraction of the melt pool in the adjacent scanning channel caused the further accumulation of stress in the scanning channel, leading to crack formation.

When the scanning spacing was small, the overlapping region caused the coating to remelt twice, which improved the flatness of the coating. Moreover, the remelting zone’s structure remelted and condensed, promoting grain refinement and further releasing residual stress. Reducing the scanning spacing improved the flatness of the coating and inhibited crack formation. However, a scanning spacing that is too small results in severe diffusion of the coating elements.

**Figure 6:**
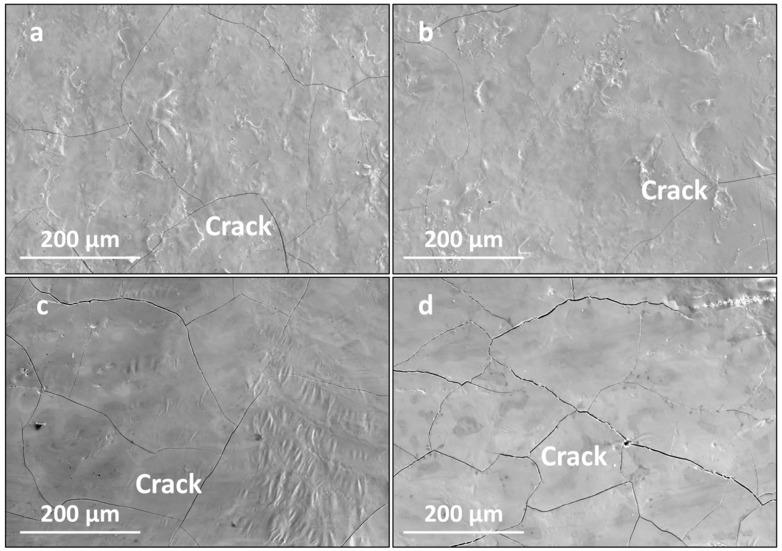
Surface morphology of the coating under different laser powers: (**a**) 27 W; (**b**) 30 W; (**c**) 33 W; and (**d**) 36 W.

Figure 6 presents the surface morphologies of the LMM coatings at different laser powers. With an increase in the laser power, the coating flatness and overlap rate were significantly optimized. When the power was low, irregular depressions and folds appeared on the coating surface, along with droplet aggregation phenomena and fine cracks. As the laser power increased, the coating morphology became flatter. However, the cracks became larger and wider, and penetrating cracks appeared. During laser processing, the laser power affects the temperature of the melt pool. A higher molten-pool temperature corresponds to a lower liquid-phase viscosity and better fluidity. Thus, high-power LMM results in flat coatings with a high tissue density [32]. However, when the power is too high, the internal temperature of the melt pool is too high, and the temperature gradient and cooling rate increase, which increases the internal stress of the coating, leading to cracks in the coating. At a lower power, less liquid phase is generated, and the temperature of the melt pool is lower, which leads to a higher viscosity and lower fluidity of the liquid phase. Moreover, the temperature decreases rapidly after laser scanning, which makes it impossible for the melt pool to spread smoothly, resulting in defects such as craters, wrinkles, and microcracks on the surface of the coated layer after the LMM treatment.

**Figure 7:**
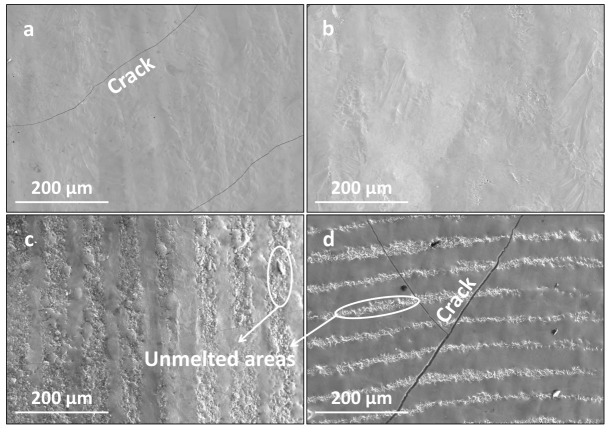
Surface morphologies of the coating at different scanning speeds: (**a**) 800 mm/min; (**b**) 1200 mm/min; (**c**) 1600 mm/min; and (**d**) 2000 mm/min.

Figure 7 presents the surface morphologies of the LMM coatings at different scanning speeds. As the scanning speed increased, the coating flatness decreased, and the cracks in the coating became larger. The heat received by the melt pool on the coating decreased, reducing the fluidity of the melt pool, which reduced the flatness of the coating and led to the formation of irregular craters and wrinkles. This was particularly noticeable at high scanning speeds. In addition, as the scanning speed increased, the temperature gradient and cooling rate of the melt pool increased, which increased the coating stress and led to the cracking of the coating. The cooling and solidification processes occurred within a short time, leaving some of the bubbles in the melt pool with no time to rise to the surface and remain in the remelted layer. Therefore, the defects in the coating were mainly in the form of pores and micropores. These defects often move and aggregate and are highly susceptible to becoming a source of crack generation. Increasing the scanning speed reduces the temperature of the melt pool, leading to an abatement in the liquid phase fluidity, which causes the liquid phase remaining between the crystals to circulate poorly, resulting in liquid metal solidification and a contraction of the liquid phase without sufficient liquid phase to replenish it. As the temperature continues to decrease, the intergranularity becomes prone to cracking [33]. The increase in the scanning speed inhibits grain growth, facilitates grain refinement, and reduces the coating stress. However, this increases the temperature gradient due to the smaller heat-affected zone and lower melt pool temperature. Differences in the thermophysical properties of the coating materials lead to larger deformation during condensation, causing the cracking of the coating.

**Figure 8:**
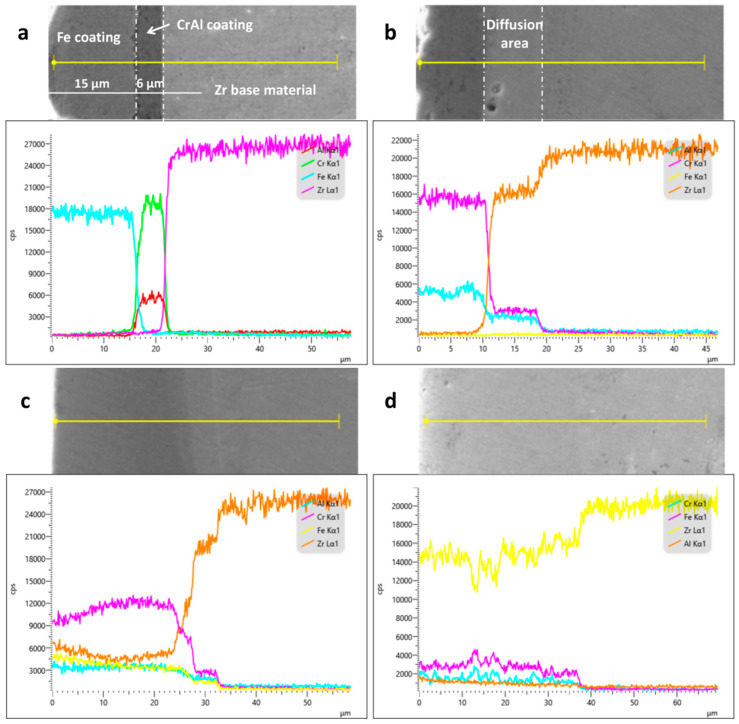
Cross-sectional morphologies of the specimens and the degrees of elemental diffusion: (**a**) before LMM treatment; (**b**) at a power density of 3.8 × 10^3^ W/mm^2^; (**c**) at a power density of 4.2 × 10^3^ W/mm^2^; and (**d**) at a power density of 4.6 × 10^3^ W/mm^2^.

The cross-sectional morphologies and elemental diffusion of the specimens subjected to the different processes are shown in Figure 8. Figure 8a shows the cross-sectional EDS line scan and micromorphology of the coating without laser processing. There is a clear demarcation line between the Fe and CrAl coatings and the Zr substrate. Comparing the EDS spectra reveals that Fe, Cr, Al, and Zr fell off at the demarcation interface in the form of cliffs, and there was no obvious diffusion of any of these elements. The thicknesses of the Fe and CrAl coatings were approximately 15 and 6 μm, respectively. As shown in Figure 8b, the boundary between the CrAl coating and the Zr substrate began to fade, and Zr melted and diffused but did not diffuse throughout the coating, because of the small amount of melted Zr. This process resulted in a metallurgical bond between the coating and substrate, which enhanced the coating’s properties. When the laser power density was increased to 4.2 × 10^3^ W/mm^2^, the demarcation line between the coating and the substrate was blurred and faded, and elemental Zr diffused throughout the coating, which led to the failure of the protective properties of the coating. With a further increase in the power density, the film–base bonding demarcation line disappeared. With an increase in the amount of the Zr matrix melted, the Zr content in the coating increased. According to the temperature field simulation analysis, the depth of the melt pool of the Zr matrix increased with an increase in the laser power and a reduction in the scanning speed, resulting in different degrees of Zr diffusion in the coating.

**Figure 9:**
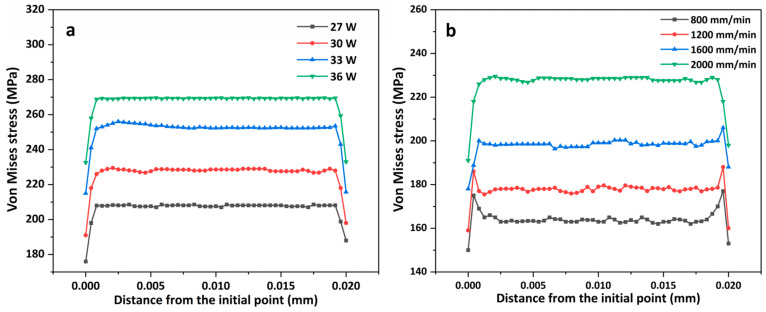
Distribution of von Mises stresses along the laser scanning path: (**a**) different powers; (**b**) different scanning speeds.

Figure 9a shows the distribution of the von Mises stresses along the laser scanning path after the specimen was cooled for 500 s at different powers. The coating residual stress increased with the laser power; however, when the laser power increased, the growth rate decreased. The coating was melted through laser irradiation to produce a liquid phase; however, the rapid cooling of the melt pool led to a sharp increase in the stress in the coating [34], which increased the risk of cracking. The accumulation of residual stress during the multipass LMM process can easily lead to the cracking of hard coatings [35]. As the power increases, the temperature gradient generated by the absorption of laser energy by the coating and the cooling rate of the coating also increase, increasing the amount of coating deformation and thus the stress. This increases the risk of the coating cracking. Moreover, because of the difference in the coefficient of thermal expansion between the material and the substrate, the stresses propagate within it as it cools, and the residual stresses become significant, causing the plasticity of the material interface to deteriorate. Cracks also occur after the material reaches its strength limit. Figure 9b shows the distribution of von Mises stresses along the laser scanning path at different scanning speeds after the specimen was cooled for 500 s. Compared with the laser power, the scanning speed had a more significant impact on the residual stresses. The cooling rate derived from the simulation of the cooling rate of the temperature field was affected more by the scanning speed, which indicates that an increase in speed augments the coating’s residual stresses, making the coating prone to cracks and other defects. The cooling rate increases with the scanning speed, and, although a high degree of subcooling can refine the grains, it can also lead to nonuniform nucleation of the grains and form coating defects. Residual stresses tend to accumulate more readily at the defects, and cracking occurs when the residual stresses exceed the strength limit of the coating.

According to the simulation and experimental results, a laser power of 30 W, scanning speed of 1200 mm/min, and scanning spacing of 0.035 mm, were selected to prepare the coating. The coating morphology was observed and its properties were tested.

**Figure 10:**
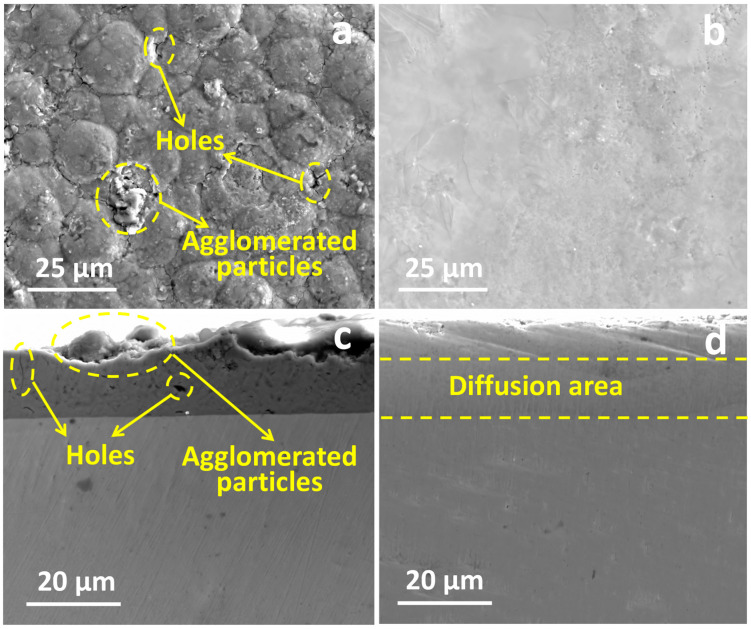
(**a**,**b**) are the surface morphology of the original and LMM coatings, respectively; (**c**,**d**) are the cross-section morphology of the original and LMM coatings, respectively.

**Figure 11:**
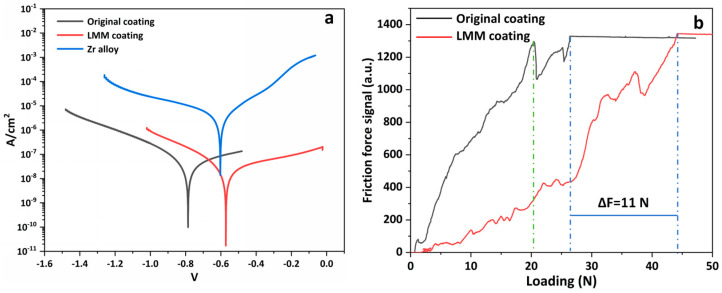
(**a**) Polarization curves; (**b**) scratch test friction force signal diagram.

Figure 10 shows the surface and cross-section morphology of the original and LMM coatings, respectively. It can be observed that the surface of the original coating is rough and uneven, with many holes and agglomerated particle defects. The demarcation line between the coating and the substrate is visible. After the LMM treatment, the coating is flat and crack-free, and the demarcation line between the coating and the substrate is blurred due to the mutual diffusion of the coating and substrate elements. Electrochemical corrosion tests were performed on different specimens, and Figure 11a shows the dynamic potential scanning polarization curves. Compared to Zr alloy substrates, the corrosion current densities of the pristine and LMM coatings decreased by two and three orders of magnitude, respectively. The corrosion resistance of the specimens was superior to that of the Zr substrate. Alloying is an effective method for making metals more corrosion-resistant. Generally, corrosion-resistant alloys composed of two metals are single-phase solid-solution alloys with high chemical stability and a resistance to corrosion in certain media. The presence of Cr, Al, and other corrosion-resistant elements in the coating increases the electrode potential of the Fe-based solid solution, which improves the corrosion resistance of the coating [36,37]. Compared with the original coating, the corrosion resistance of the LMM coating was improved by only one order of magnitude. The coatings prepared via the LMM process had good morphologies, and defects such as the porous droplets caused by the electroplating and PVD processes were eliminated. The improvement of the coating’s flatness directly reduces the contact area of the coating in corrosion, which in turn reduces the damage to the coating of the corrosive fluid. At the same time, the elimination of defects such as holes cuts the corrosive fluid off from the internal channels of the coating, which in turn helps to improve the corrosion resistance of the coating. However, because of the edge effect, the stresses accumulated in the edge region of the specimen were higher, leading to microscopic cracks. This weakened the protective properties of the coating. The specimens before and after the LMM treatment were tested for scratch bonding. The results of the scratch test are presented in Figure 11b, where the point at which the curve undergoes a sudden change corresponds to the critical load of the coating. The coating without the LMM treatment exhibited two abrupt changes, corresponding to the critical loads of the Fe and CrAl coatings. The friction signals of the coatings after the LMM treatment exhibited only one abrupt change, indicating that the Fe coating amalgamated well with the CrAl coating, which is consistent with the simulation results, EDS results, and cross-section morphology analysis. The film–base bonding force was increased by 56% after the LMM treatment. The coating and substrate diffuse into each other, transforming the mechanical bond into a metallurgical bond with a higher strength. Simultaneously, the intermelting between the coating and the base element effectively reduces the negative effects of the differences in the thermophysical properties between different elements in the high-temperature environment. This allowed the coating to maintain its excellent adhesion properties under thermal shock.

## 4. Conclusions

By combining the advantages of PVD and electroplating, an FeCrAl coating was pre-positioned on the surface of a Zr alloy, which was modified via an LMM treatment. The temperature and stress fields generated in the LMM process were analyzed using finite element simulations to narrow down the selection of the process parameters. Additionally, the effects of the LMM process parameters on the coating morphology were investigated. The results indicated that the laser power significantly affected the coating morphology and degree of elemental diffusion. Higher scanning speeds were more likely to cause stress accumulation in the coating, and the remelted coating overlapped poorly. Reducing the offset improved the coating flatness and effectively suppressed crack formation. With the LMM treatment, the coating and matrix elements diffused into each other, the film–base demarcation line disappeared, and a metallurgical bond was formed. At a laser power of 30 W, scanning speed of 1200 mm/min, and scanning spacing of 0.035 mm, the coating morphology was good and defects existing in the original coating were eliminated. Compared to the original coating, after the LMM treatment, the coating’s film–base bonding force increased by 56% and its corrosion resistance was improved. A corrosion-resistant coating with a strong bonding force was successfully prepared. This investigation provides valuable guidance for improving the laser modification of micron-sized protective coatings.

## Figures and Tables

**Table 1:** PVD process parameters.

Substrate Bias Voltage	Target Current	Deposition Time	Ar Gas Flow Rate	Duty Cycle	Target Base Distance	Substrate Temperature
−200 V	65 A	90 min	110 mL/min	50%	170 mm	300 °C

**Table 2:** Composition of the plating solution.

ChCl	EG	FeCl_2_·4H_2_O
45 g	40 mL	67.5 g

**Table 3:** Electroplating process parameters.

Plating Solution Temperature	Current Density	Plating Time	Activation Time
70 °C	45 mA/cm^2^	20 min	25 s

**Table 4:** Thermophysical performance parameters.

Thermophysical Performance Parameters of CrAl (Melting Point: 1154 °C)							
**Temperature (°C)**	20	300	600	900	1154	1220	1700
**Specific heat (J/(kg·°C))**	596	720	754	804	870	10,702	991
**Thermal conductivity (W/(m·°C))**	27.3	34.3	38.9	42.6	45.6	31.2	33.8
**Density/(Kg/m^3^)**	4933	4843	4716	4594	4475	4313	4030
**Thermophysical Performance Parameters of Fe (Melting Point: 1538 °C)**							
**Temperature (°C)**	20	600	1000	1538	1556	1566	1800
**Specific heat (J/(kg·°C))**	452	790	711	739	9416	826	827
**Thermal conductivity (W/(m·°C))**	50.5	33.8	30.3	35.5	33.7	34.1	38.6
**Density/(Kg/m^3^)**	7798	7603	7447	7220	6930	6914	6708
**Thermophysical Performance Parameters of Fe (Melting Point: 1825 °C)**							
**Temperature (°C)**	20	600	1200	1700	1820	1825	2000
**Specific heat (J/(kg·°C))**	286	377	325	987	16,185	458	460
**Thermal conductivity (W/(m·°C))**	17.4	20.2	28.6	37.8	37.1	36.4	39.5
**Density/(Kg/m^3^)**	6558	6482	6413	6207	5966	5836	5742

**Table 5:** Mechanical performance parameters.

Mechanical Performance Parameters of Zr Alloys (Yield Strength: 530 MPa)							
**Temperature (°C)**	25	400	800	1200	1600	1820	2000
**Average expansion coeff (10^−6^·K^−1^)**	5.78	6.41	6.83	6.43	8.42	18.39	24.37
**Young’s modulus (GPa)**	70	58	48	70	64	1	1
**Poisson’s ratio**	0.32	0.32	0.32	0.32	0.32	0.44	0.49
**Mechanical Performance Parameters of FeCrAl (Yield Strength: 595 MPa)**							
**Temperature (°C)**	25	200	400	600	800	1000	1530
**Average expansion coeff (10^−6^·K^−1^)**	11.04	11.76	12.62	13.47	14.33	15.18	17.36
**Young’s modulus (GPa)**	211	200.7	183.5	160.9	134.8	107.6	1
**Poisson’s ratio**	0.30	0.30	0.31	0.32	0.33	0.34	0.49

## Data Availability

Data are contained within the article.
